# Genetic Variations and mRNA Expression of Goat *DNAH1* and Their Associations with Litter Size

**DOI:** 10.3390/cells11081371

**Published:** 2022-04-18

**Authors:** Zhen Wang, Ruolan Wang, Chuanying Pan, Hong Chen, Lei Qu, Lian Wu, Zhengang Guo, Haijing Zhu, Xianyong Lan

**Affiliations:** 1 Lab of Animal Genome and Gene Function, College of Animal Science and Technology, Northwest A&F University, Xianyang 712100, China; wangzhenid@126.com (Z.W.); wangruolan7@126.com (R.W.); chuanyingpan@126.com (C.P.); chenhong1212@263.net (H.C.); lxw784@alumni.bham.ac.uk (L.W.); gzg0857@163.com (Z.G.); 2 Shaanxi Provincial Engineering and Technology Research Center of Cashmere Goats, Yulin University, Yulin 719000, China; ylqulei@126.com; 3 Life Science Research Center, Yulin University, Yulin 719000, China; 4 Shaanxi Province “Four Subjects One Union” Sheep and Goat Engineering & Technology University & Enterprise Alliance Research Center, Yulin 719000, China

**Keywords:** goat, *DNAH1*, SNP, InDel, CNV, litter sizes

## Abstract

*Dynein Axonemal Heavy Chain 1* (*DNAH1*) encodes proteins which provide structural support for the physiological function and motor structure of spermatozoa (hereafter referred to as sperm) and ova. This study found that three single nucleotide polymorphisms (SNPs), the 27-bp insertion/deletion (InDel) mutations and three exonic copy number variations (CNVs) within *DNAH1* were significantly associated with litter size of Shaanbei white cashmere goats (*n* = 1101). Goats with the wildtypes of these three SNPs had higher litter sizes than other carriers (*p* < 0.05). II genotype of the 27-bp InDel had the highest litter size compared with ID carriers (*p* = 0.000022). The gain genotype had the largest litter sizes compared with the loss or medium carriers for the three CNV mutations (*p* < 0.01). Individuals with the AA-TT-CC-II-M_1_-M_2_-M_3_ and AA-TT-CC-II-G_1_-G_2_-M_3_ combination genotypes had larger litter sizes compared with the other genotypes. This study also showed the *DNAH1* expression in mothers of multiple kids was higher than mothers of single kids. These three SNPs, the 27-bp InDel and three CNVs in *DNAH1* could be used as molecular markers for the selection of goat reproductive traits.

## 1. Introduction

The Shaanbei white cashmere goat (SBWC) excels in the production of both fine cashmere and meat, and therefore plays an important role in the Chinese goat industry. Nevertheless, there is much scope for improvement in their reproductive performance. Poor reproductive performance in goat breeds has impeded the growth of the goat industry [1,2]. Hence, there is a pressing need to develop effective and practical measures for improving reproductive traits in this species [3].

Some animal phenotypes are qualitative traits with high heritability, but most traits associated with reproduction are not. They are often of low heritability, and methods of traditional breeding selection are often not suitable for improving these traits [4]. In contrast, modern molecular labeling techniques can be used to identify beneficial genes, the selection of which will assist in breeding excellent individuals [5]. Some known molecular mutations, single nucleotide polymorphism (SNP) mutations [6], insertion/deletion (InDel) and copy number variant (CNV) variations can be identified quickly and have been employed as useful and efficient molecular markers in the breeding of goats and other livestock. Indeed, SNP, InDel and CNV have been widely used to screen for reproduction-related candidate genes [3,7,8]. Meanwhile, the relationships between SNP, InDel and CNV mutations within certain genes have not been explored yet. Here, we want to explore some mutations within candidate genes and detect whether SNP, InDel and CNV mutations of it are correlated. We also want to identify the mRNA expression of a candidate gene in ewes with single kids and multiple kids. Specifically, we will study the relationship between this gene for reproduction and goat litter size by exploring the associations of SNP, InDel and CNV mutations.

*Dynein Axonemal Heavy Chain 1* (*DNAH1*) encodes proteins which provide structural support for the physiological function and motor structure of spermatozoa (hereafter referred to as sperm) and ova [9,10]. Some genetic mutations within *DNAH1* lead to multiple morphological anomalies of the flagella (MMAF) [11], and clinically, MMAF is often accompanied by abnormal sperm morphology and function, leading to male infertility. Many morphological and structural defects can be identified in sperm such as short, curved, irregular morphology and even a complete loss of flagella [12]. Furthermore, abnormal sperm morphology has been documented to affect reproduction and cause infertility in humans and mice [13], dogs [14] and cattle [15]. Meanwhile, the *DNAH1* gene is also associated with primary ciliary dyskinesia (PCD) [16,17]. PCD is a type of autosomal recessive inheritance caused by defects in cilia structure or function that are induced by gene mutation. It can seriously affect both male and female reproductive capacity [16]. In males, the sperm tail is a cilia variant so an abnormal structure could cause it to lose motility, which may lead to infertility. In females, the surface of the fallopian tubes is covered by a rhythmically oscillating cilia which ensures the transport of ova; thus, structural dysfunction of cilia can lead to ectopic pregnancy and even infertility [18,19].

As an MMAF-related gene, *Cilia* *and flagella associated protein 43* (*CFAP43)* gene has been reported to significantly affect kidding traits in goats [20]. Meanwhile, *DNAH1* belongs to the MMAF-related gene, and it is a reproductive candidate gene that is associated with both female and male gonadal development and fertility. The *DNAH1* gene encodes an inner dynein arm heavy chain that provides structural support between the radial spokes and the outer doublet of the sperm tail, and females can exhibit sub-fertility owing to defective oviduct cilia. Additionally, *DNAH1* is also broadly expressed in the testis, ovary and placenta [13,21]. To date, there has been no evidence to confirm whether this gene is related to reproductive traits in goats. Based on the aforementioned studies, we hypothesized that *DNAH1* was linked to goat reproductive traits. The objectives of this study were to identify genetic SNP, InDel and CNV polymorphisms in the *DNAH1* gene in SBWC goats, as well as explore the difference in *DNAH1* expression between mothers of single kids and mothers of multiple kids. We suggest that the results of this study could be used in the design of effective goat breeding programs that would promote growth within the goat industry.

## 2. Materials and Methods

### 2.1. DNA Isolation and Total RNA Extraction form Goat Tissues

For DNA extraction, a total of 1101 adult, female, physically mature SBWC goats were randomly selected from an SBWC farm in Shaanxi Province, China. For RNA extraction, a total of 6 adult female goats were selected, including 3 mothers of single kids and 3 mothers of multiple kids. All does were of similar age and weight and raised under the same external environment [20,22]. After weaning, does were all fed a diet of corn, wheat bran, soybean meal and premix. Farm workers were responsible for the raising and management of all experimental goats and none showed any adverse health conditions. On the SBWC goat farm, veterinarians recorded the number of first litter per goat on a daily basis. The phenotypic distribution of does with different litter sizes is shown in Table 1. Individuals used for sampling were randomly selected from the goat farm to ensure that they were unrelated to each other [7].

According to our previous report, we collected doe ear tissue and ovary samples before the goats were sold or slaughtered. After this sampling step was completed, all samples were placed in an alcohol solution and immediately frozen. The process of sample collection and treatment and the steps of genome-wide extraction were performed as previously reported [20,23]. The quality and purity of each DNA sample was measured by Nanodrop 1000 (Thermo Fisher Scientific Inc., Wilmington, DE, USA). According to previous studies, the mass concentration of DNA extracted from each sample was high [24]. Then, DNA samples were diluted with ddH_2_O to a standard concentration of 20 ng/uL and stored at −20 °C for subsequent genotyping. For RNA assay, in total 6 adult female goats were selected including 3 mothers of single kids and 3 mothers of multiple kids. According to our previous report, we collected doe ear tissue and ovary samples before the goats were slaughtered. Total RNA isolation of ovary and synthesis of cDNA were shown as before [2].

### 2.2. Identification of Candidate Mutations and Primer Design

**Figure 1:**
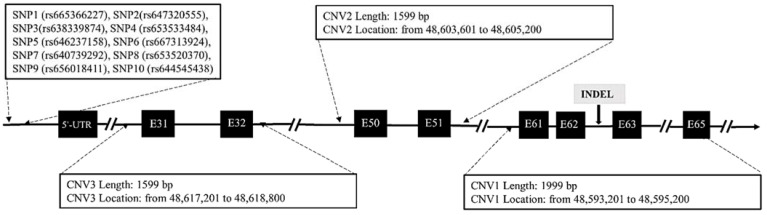
Genome map that indicates the location of the SNP, InDel and CNV mutations in goat *DNAH1* gene.

For SNP detection, using the Ensembl online database (http://asia.ensembl.org/, accessed on 1 December 2020), a pair of primers were designed to amplify fragment containing 10 SNP mutations within the promoter region of *DNAH1* gene in 327 goats. These variations including SNP1 (rs665366227), SNP2(rs647320555), SNP3(rs638339874), SNP4 (rs653533484), SNP5 (rs646237158), SNP6 (rs667313924), SNP7 (rs640739292), SNP8 (rs653520370), SNP9 (rs656018411) and SNP10 (rs644545438). For InDel detection, a total of 9 putative InDel mutations of *DNAH1* were studied (P1–P9). In accordance with a previous prediction, the corresponding primer sequences were designed according to the 9 mutation positions using Primer software 5.0 (Canada, Premier). For CNV detection, validation of goat exonic CNV1 (g:48593201–48595200), CNV2 (g:48603601–48605200) and CNV3 (g:48617201–48618800) within the *DNAH1* gene in chromosome 22 was performed referring to the Animal Omics Database (http://animal.nwsuaf.edu.cn/, accessed on 10 July 2021). Three pairs of primers were designed to amplify these three CNV regions of *DNAH1* in 318 goats (Figure 1). Given that previous studies suggest that there are neither CNVs nor segmental duplication in the goat melanocortin 1 receptor (*MC1R*) gene, we selected *MC1R* as the internal reference gene and designed a pair of primers to amplify this target sequence [25]. The specific primer sequences are shown in Table S1.

### 2.3. Detection of SNP, InDel and CNV Mutations of DNAH1 Gene

**Figure 2:**
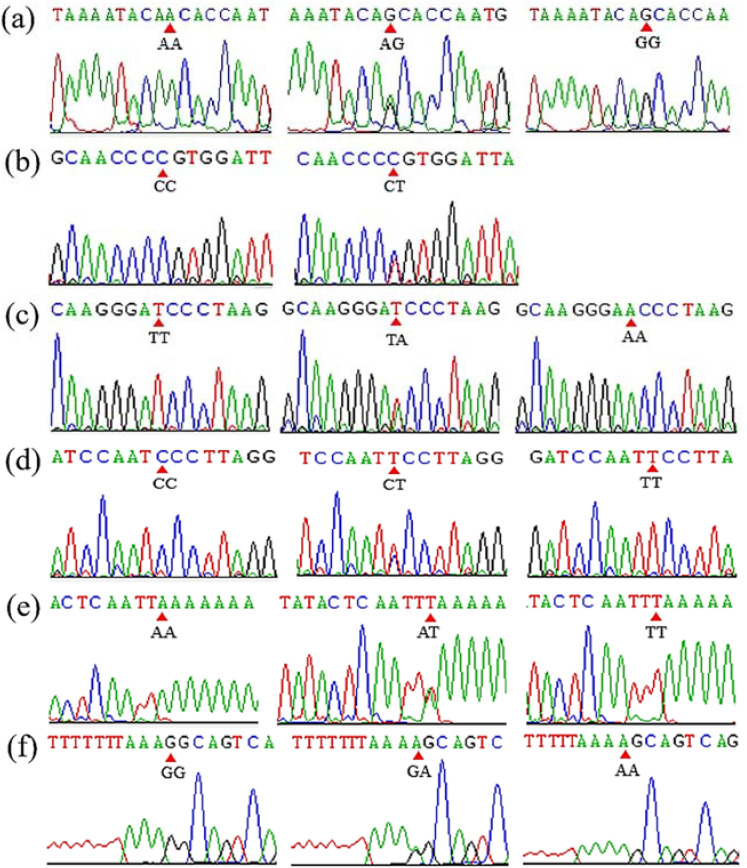
The result of sanger sequencing of SNP1 (**a**), SNP4 (**b**), SNP5 (**c**), SNP7 (**d**), SNP9 (**e**) and SNP10 (**f**) mutations in goat *DNAH1* gene.

**Figure 3:**
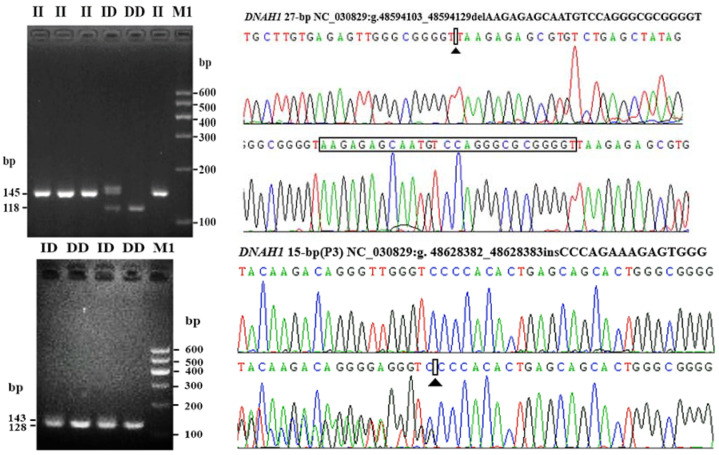
Electrophoresis and sequence diagrams of P1 and P3 InDel loci of *DNAH1* gene in Shaanbei white cashmere goat. The P1 locus is the result of II and DD genotype sequencing. P3 is the DD and ID genotype sequencing result.

Mutant sites that proved to be polymorphic were further identified in the experimental goats. By the Sanger sequencing, 10 SNP mutations were identified according to whether they were polymorphisms. The sequencing results are shown in Figure 2. Two putative InDel mutations of *DNAH1* (*DNAH1*-P1 and *DNAH1*-P3) were verified using traditional PCR methods, as previously reported [20,22]. Gel electrophoresis and Sanger sequencing results of the *DNAH1*-P1 and *DNAH1*-P3 InDel loci are shown in Figure 3. For the three CNV regions within *DNAH1* and the internal reference *MC1R*, real-time quantitative PCR (qPCR) was used to amplify the target fragment using SYBR Green reactions in triplicate. The reaction volume was 10 μL, and each reaction contained 16 ng of genomic DNA and 5 μL of SYBR Green Mix (BIOER, Hangzhou, China). Thermal cycling conditions were as follows: 95 °C for 10 min followed by 40 cycles of 95 °C for 15 s, 60 °C for 30 s and 72 °C for 20 s.

### 2.4. Detection of mRNA Expression of DNAH1 Gene

For the detection of *DNAH1* expression assay, the cDNA of both sides of ovary samples including 3 mothers of single kids and 3 mothers of multiple kids were used to explore the mRNA expression of *DNAH1* gene by qPCR method. The primers of qPCR and internal gene are shown in Table S1. The procedure was the same as the procedure of CNV method.

### 2.5. Statistical Analyses

As previously reported, the population indexes of the experimental goats were calculated [26], including genotype frequency, allele frequency, homozygosity (Ho), heterozygosity (He), polymorphism information content (PIC), etc. In linkage disequilibrium (LD) analysis, the case of r^2^ < 0.33 was considered to represent weak LD; r^2^ > 0.33 was considered to represent strong LD; and r^2^ = 1 was considered to represent complete LD [27]. The relationships between SNP, InDel and CNV mutations and goat litter sizes were analyzed using chi-square analysis of non-parametric methods and a single-factor analysis method.

In the manuscript, the following fixed model was used: statistical model applied to determine the association of litter sizes with different mutation genotypes: Y*_ijklm_* = μ + G*_i_* + B*_j_* + F*_k_* + A*_l_*+ e*_ijklm_*, where Y*_ijklm_* is the phenotypic value of litter size trait, μ is the population mean, G*_i_* is the fixed effect of genotype, B*_j_* represents age effect, F*_k_* is herd effect, A*_l_* stands for regional effect and e*_ijklm_* is the random error [20,28]. Compared with the internal reference gene, the analysis method of copy number variation in each sample: ΔCt = Ct_target gene_ − Ct_reference gene,_ cycle threshold (Ct) was used to represent the mean of triplicate independent individuals for CNV mutation detection [2,3]. Finally, genotypes of CNV mutations within *DNAH1* were divided into three types, including loss (<2), medium (=2) and gain (>2). Experiments were repeated three times and the mean value of intensity ratios ± standard error (SE) was shown [29,30].

The 2^−ΔΔCt^ method and *t*-test were used to analyze the expression difference of *DNAH1* gene in ovary [31].

## 3. Results

### 3.1. Identification of SNP, InDel and CNV Polymorphisms within the DNAH1 Gene

We identified SNP1 (rs665366227), SNP3(rs638339874), SNP5 (rs646237158), SNP7 (rs640739292), SNP9 (rs656018411) and SNP10 (rs644545438) were polymorphic (Table 2 and Figure 2). After using agarose gel electrophoresis and Sanger sequencing, the results of InDel mutation are shown in Table 2 and Figure 3. The information associated with the two InDel mutations were as follows: the 27-bp InDel (NC_030829.1:rs636295440; g.48594103_48594129delAAGAGAGCAATGTCCAGGGCGCGGGGT) was identified using primer 1, whereas the 15-bp InDel (NC_030829.1: rs662697370; g.48628382_48628383ins CCCAGAAAGAGTGGG) was identified using primer 3. Three exonic CNV loci were identified within the exonic regions of *DNAH1*: CNV1 (NC_030829.1, Chr22:48593201–48595200, 1999 bp), CNV2 (NC_030829.1, Chr22:48603601–48605200, 1599 bp), and CNV3 (NC_030829.1, Chr22:48617201–48618800, 1500 bp). We also found that the 27-bp InDel (*DNAH1*-P1) was located inside the CNV1 region of *DNAH1*.

### 3.2. Allele and Genotype Frequencies of SNP, InDel and CNV Variations in DNAH1 Gene

The results of allele and genotype frequencies of SNP1 (rs665366227), SNP3 (rs638339874), SNP5 (rs646237158), SNP7 (rs640739292), SNP9 (rs656018411) and SNP10 (rs644545438) are shown in Table 2. The χ^2^ test indicated that the genotypic distributions of SNP1, SNP4, SNP5, SNP7, SNP9 and SNP10 were all consistent with Hardy–Weinberg equilibrium (*p* > 0.05). Agarose gel electrophoresis showed that there were three genotypic variants of the 27-bp InDel (*DNAH1*-P1): homozygotic insertion, heterozygote and homozygotic deletion type were replaced by II, ID and DD, respectively. In the goat population, we only found a single DD carrier. For the 27-bp InDel site, the “I” allele was associated with the main allele in goat individuals. Meanwhile, the 15-bp InDel (*DNAH1*-P3) had two genotypes; there were II and ID carriers. Results showed the “D” allele was associated with the dominant allele in goat individuals. The χ^2^ test indicated that the genotypic distributions of these two InDels were in Hardy–Weinberg equilibrium (*p* > 0.05; Table 2). For these three exonic CNVs of *DNAH1*, the results showed that all three genotypes were present, including loss, medium and gain types.

### 3.3. Association Analysis between SNP, InDel and CNV Variations with Goat Litter Size

Association analysis illustrated that SNP1 (rs665366227), SNP5 (rs646237158) and SNP7 (rs640739292) were significantly associated with goat litter sizes (*p* < 0.05), and wild genotypes had higher litter sizes than heterozygous genotypes and homozygous mutant genotypes (Table 3). The 27-bp InDel within *DNAH1* was significantly related to goat litter size (*p* = 0.000022), and the litter sizes of II carriers were higher than those of ID carriers. However, the 15-bp InDel was not related (*p* = 0.605; Table 3). For exon CNV1, the gain type had significantly larger litter sizes than the medium and loss types. The medium type had significantly higher litter sizes than the loss types (*p* = 0.000011). For exon CNV2, the gain type had significantly larger litter sizes than the other two types (*p* = 0.003). For exon CNV3, the gain type also had significantly larger litter sizes than the medium and loss types (*p* = 0.000187; Table 4).

### 3.4. Relationship between Combination Genotypes and Goat Litter Size

*DNAH1*-SNP1, *DNAH1*-SNP5, *DNAH1*-SNP7, *DNAH1*-P1, *DNAH1*-CNV1, *DNAH1*-CNV2 and *DNAH1*-CNV3 were all associated with goat litter size (*p* < 0.05). Next, we aimed to explore the relationship between combinations of genotypes and goat litter size. Among 1101 individuals, we selected combinations of genotypes with sample sizes greater than three for association analysis. There were eight types of combination genotypes (SNP1-SNP5-SNP7-InDel-CNV1-CNV2-CNV3): AA-TT-CC-II-L1-L2-L3, AA-TT-CC-II-M1-L2-L3, AA-TT-CC-II-M1-M2-M3, AA-TT-CC-II-G1-G2-M3, AG-TA-CT-II- L1-L2-L3, AG-TA-CT-II- M1-M2-L3, AG-TA-CT-II- M1-G2-M3 and GG-AA-TT-II- L1-L2-L3. Furthermore, single-factor analysis revealed that the combination genotype 3 (AA-TT-CC-II-M1-M2-M3) and combination genotype 4 (AA-TT-CC-II-G1-G2-M3) carriers had the largest litter sizes among all combination genotypes (Figure S1). Furthermore, we found that there was strong LD between SNP1, SNP5 and SNP7 mutations (r^2^ > 0.33; Figure S2).

### 3.5. mRNA Expression of DNAH1 in Ovary of Goats

The qPCR results proved the mRNA expression of the *DNAH1* gene was different in mothers of single kids and mothers of multiple kids. The mRNA expression showed that the *DNAH1* gene had higher expression in mothers of multiple kids than mothers of single kids. Although there is no significant difference between them (*p* > 0.05), the relationship between mRNA expression of goat *DNAH1* gene and litter size traits deserve to be further explored. This result indicates that *DNAH1* expression might play an important role in litter size traits.

## 4. Discussion

The *DNAH1* gene is broadly expressed in multiple reproductive tissues, including the testis, ovary and placenta, and is therefore a candidate gene for reproductive traits [21,32]. According to previous reports, mutations within the *DNAH1* gene are associated with phenotypes of MMAF and PCD [13,18]. MMAF has been widely studied in male reproductive traits and is often associated with abnormal sperm, which seriously affects male reproductive traits and even leads to infertility [12]. Indeed, as an MMAF-related gene, many papers have identified *DNAH1* as playing an essential role in the development of male and female gonads in humans and have explored the clinical applications of *DNAH1* [33]. PCD is often accompanied by cilia movement disorder, which is also an autosomal recessive genetic disease. Patients with severe PCD exhibit infertility. Clinically, females can exhibit sub-fertility owing to defective oviduct cilia; on the other hand, males can be infertile because of immotile sperm flagella [17,34,35]. Imtiaz et al. speculated that females may be more sensitive to severe variants of *DNAH1* and that these variants may have more severe effects than those identified in males [18]. Specifically, they found that molecular variation in *DNAH1* may play a role in PCD, which suggests that its potential contribution to reproduction should be considered in future studies.

Abnormal sperm morphology has been documented to affect reproduction and cause infertility in humans and mice [13], dogs [14] and cattle [15]. We have found that an InDel variation within an MMAF-associated gene, *CFAP43* gene, significantly affected litter size in goats [20]. This suggests that MMAF-related genes can also influence reproductive performance in goats. *DNAH1* gene encodes an inner dynein arm heavy chain that provides structural support between the radial spokes and the outer doublet of the sperm tail, and females can exhibit sub-fertility owing to defective oviduct cilia. Mice with a homozygous knockout of the orthologous gene are viable but have reduced sperm motility and are infertile.

**Figure 4:**
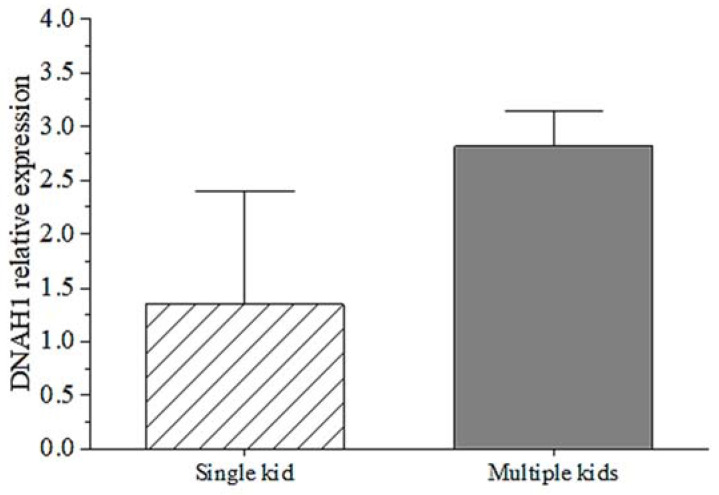
The mRNA expression of goat *DNAH1* gene in ovarian tissues of mothers of single kids and mothers of multiple kids.

There are only some reports regarding the relationship between the *DNAH1* gene and the reproductive performance of humans and mice. In this study, we found *DNAH1* expression of mothers of multiple kids was higher than mothers of single kids (Figure 4). This indicated that the *DNAH1* gene might play an essential role in goat productive traits. The current study explored the relationship between several genetic variants and goat litter size to provide scientific guidance for the breeding of goat reproductive traits. In this study, three SNPs, a 27-bp InDel and three exonic CNV loci within *DNHA1* were all associated with goat litter size traits (*p* < 0.05; Table 4). These results suggest that these three SNPs, the 27-bp InDel and the three exonic CNV mutations of *DNAH1* would be of great value in the genetic selection of goat reproductive traits. Some studies reported that genetic polymorphisms could affect gene expression and, consequently, some growth and reproduction traits of animals [36,37] and humans [38,39]. For example, there is a single nucleotide mutation in the *IGF2* gene of the Bama pig, which can significantly affect skeletal muscle development in this breed. Through genetic breeding of this mutation, the weight traits of this pig breed can be significantly improved [40]. Likewise, we found that these SNP, InDel, and CNV sites within *DNAH1* could significantly affect goat reproductive traits, suggesting that these are important variants with potential value in breeding programs owing to their collective action in the modulation of reproductive traits.

Previous reports identify that linkage relationships between different mutation sites can exert a synergistic effect on the phenotypic traits of livestock [41,42]. Considering that these three SNPs, the 27-bp InDel and the three CNVs simultaneously affect goat litter size, and the relationship between SNP, InDel and CNV mutations has not been previously reported. We speculated on the existence of an association between these SNPs, InDels and CNVs in *DNAH1*. In the current study, we found that these seven genetic mutations all had significant effects on goat litter size. There was a strong linkage disequilibrium between SNP1, SNP5 and SNP7 sites (r^2^ > 0.33; Figure S2), and other mutation sites may not affect goat reproduction through strong LD. Thus, the specific mechanisms underlying the functional relationships between these variants deserve to be further studied.

Given that multiple mutations of *DNAH1* can play important roles in determining litter size, we believe that these mutations may function synergistically. However, the specific functional mechanism remains to be further explored. Analysis of combined genotypes revealed that individuals with the combination genotypes AA-TT-CC-II-M_1_-M_2_-M_3_ and AA-TT-CC-II-G_1_-G_2_-M_3_ had larger litter sizes relative to other genotypes. This finding illustrates that by selecting for these three SNP sites, the 27-bp InDel and the three CNVs simultaneously, litter size traits could be improved rapidly and efficiently; however, the specific mechanism remains to be further explored.

## 5. Conclusions

In this study, three SNPs, the 27-bp InDel and three exonic CNVs within *DNAH1* significantly affected litter sizes of Shaanbei white cashmere goats, and *DNAH1* expression of ewes with multiple kids was higher than ewes with single kids. Meanwhile, individuals with the combination genotypes AA-TT-CC-II-M_1_-M_2_-M_3_ and AA-TT-CC-II-G_1_-G_2_-M_3_ had the largest litter sizes, which could be used as the effective DNA marker.

## Figures and Tables

**Table 1:** The Phenotypic Distribution of Ewes with Different Litter sizes.

Litter Size Traits	Sample Size
Mothers of single kid	550
Mothers of double kids	498
Mothers of three kids	14
Mothers of four kids	1
No record	38

**Table 2:** Genotypic Frequencies and Population Indexes for SNP and InDel Mutations of *DNAH1*.

Loci (Size)	Genotypes (Size)	Frequency		Ho	He	PIC	χ^2^ (*p*-Value)
		Genotypes	Alleles				
SNP1	AA (146)	0.559	0.736 (A)	0.611	0.389	0.313	2.294
(*n* = 261)	AG (92)	0.353	0.264 (G)				(*p* = 0.130)
	GG (23)	0.088					
SNP3	GG (234)	0.777	0.889 (G)	0.802	0.198	0.178	4.721
(*n* = 301)	GA (67)	0.223	0.111 (A)				(*p* = 0.030)
	AA (0)	0					
SNP4	CC (204)	0.872	0.932 (C)	0.873	0.127	0.119	0.864
(*n* = 234)	CT (28)	0.120	0.068 (T)				(*p* = 0.352)
	TT (2)	0.009					
SNP5	TT (124)	0.527	0.715 (T)	0.592	0.408	0.325	1.556
(*n* = 235)	TA (88)	0.374	0.285 (A)				(*p* = 0.212)
	AA (23)	0.099					
SNP7	CC (191)	0.608	0.756 (C)	0.631	0.369	0.301	0.143
(*n* = 314)	CT (93)	0.296	0.244 (T)				(*p* = 0.705)
	TT (30)	0.096					
SNP9	AA (189)	0.601	0.779 (A)	0.655	0.345	0.285	0.205
(*n* = 314)	AT (111)	0.354	0.221 (T)				(*p* = 0.651)
	TT (14)	0.045					
SNP10	GG (180)	0.583	0.754 (G)	0.629	0.371	0.302	1.746
(*n* = 309)	GA (106)	0.343	0.246 (A)				(*p* = 0.186)
	AA (23)	0.074					
27 bp	II (*n* = 920)	0.928	I (0.964)	0.930	0.070	0.068	0.0780
(*n* = 991)	ID (*n* = 70)	0.071	D (0.036)				(*p* = 0.780)
	DD (*n* = 1)	0.001					
15 bp	II (*n* = 0)	0	I (0.022)	0.958	0.042	0.042	0.169
(*n* = 346)	ID (*n* = 15)	0.043	D (0.978)				(*p* = 0.680)
	DD (*n* = 331)	0.957					

**Table 3:** Genotype Distribution of *DNAH1* Gene Indel Mutations between Mothers of Single Kids and Multiple Kids in Shaanbei White Cashmere Goat.

Loci	Sizes	MSL Genotypes			MML Genotypes			*p*-Values
		Wild Genotype	Heterozygous Genotype	Mutant Genotype	Wild Genotype	Heterozygous Genotype	Mutant Genotype	
*SNP1*	261	70	61	13	76	31	10	0.021
*SNP3*	301	129	28	0	105	39	0	0.054
*SNP4*	234	112	15	2	92	13	0	0.436
*SNP5*	235	60	58	13	64	30	10	0.041
*SNP7*	314	87	59	20	104	34	10	0.005
*SNP9*	314	104	56	6	85	55	8	0.555
*SNP10*	309	85	64	13	95	42	10	0.091
InDel-P1	991	478	56	1	442	14	0	0.000022
InDel-P3	346	220	9	0	111	6	0	0.605

MSL, mothers of single kids; MML, mothers of multiple kids (≥2).

**Table 4:** Statistical Association Analysis of Three CNVs of *DNAH1* with Litter Sizes in Goats.

Loci	Sizes	MSL Genotypes			MML Genotypes			*p*-Values
		Loss	Medium	Gain	Loss	Medium	Gain	
CNV1	296	104	38	8	66	50	30	0.000011
CNV2	301	71	37	42	46	36	69	0.003
CNV3	294	126	16	7	92	35	18	0.000187

MSL, mothers of single kids; MML, mothers of multiple kids (≥2).

## Data Availability

All Whole goat genome sequencing data were deposited in National Center for Biotechnology Information, accession number NC_030829.1 (https://www.ncbi.nlm.nih.gov/nuccore/NC_030829.1?report=genbank&from=48584957&to=48662109&strand=true) (accessed on 7 February 2022), and Ensembl, accession number HGNC:2940 (https://asia.ensembl.org/Capra_hircus/Gene/Summary?db=core;g=ENSCHIG00000013326;r=22:48585001-48662008;t=ENSCHIT00000018874) (accessed on 15 March 2022).

## Supplementary Materials

The following supporting information can be downloaded at: https://www.mdpi.com/article/10.3390/cells11081371/s1, Table S1: PCR primers used for detecting in this study; Figure S1: Relationship between the combined genotypes of SNP, the 27-bp InDel, and CNV variations within *DNAH1* gene and litter sizes in goats (mean ± standard errors); Figure S2: Linkage disequilibrium analysis of the SNP, InDel and CNV mutations within *DNAH1* in Shaanbei white cashmere goats.

## References

[1] Wang K., Kang Z., Jiang E., Yan H., Zhu H., Liu J., Qu L., Lan X., Pan C. (2020). Genetic effects of DSCAML1 identified in genome-wide association study revealing strong associations with litter size and semen quality in goat (*Capra hircus*). Theriogenology.

[2] Zhang X., Zhang S., Tang Q., Jiang E., Wang K., Lan X., Pan C. (2020). Goat sperm associated antigen 17 protein gene (SPAG17): Small and large fragment genetic variation detection, association analysis, and mRNA expression in gonads. Genomics.

[3] Bi Y., Feng W., Kang Y., Wang K., Yang Y., Qu L., Chen H., Lan X., Pan C. (2021). Detection of mRNA expression and copy number variations within the goat FecB gene associated with litter size. Front. Vet. Sci..

[4] Shaat I., Mäki-Tanila A. (2009). Variation in direct and maternal genetic effects for meat production traits in Egyptian Zaraibi goats. J. Anim. Breed. Genet..

[5] Knorst V., Byrne S., Yates S., Asp T., Widmer F., Studer B., Kölliker R. (2019). Pooled DNA sequencing to identify SNPs associated with a major QTL for bacterial wilt resistance in Italian ryegrass (*Lolium multiflorum* Lam.). Theor. Appl. Genet..

[6] Jiang E., Kang Z., Wang X., Liu Y., Liu X., Wang Z., Li X., Lan X. (2021). Detection of insertions/deletions (InDels) within the goat Runx2 gene and their association with litter size and growth traits. Anim. Biotech..

[7] Wang X., Yang Q., Wang K., Yan H., Pan C., Chen H., Liu J., Zhu H., Qu L., Lan X. (2019). Two strongly linked single nucleotide polymorphisms (Q320P and V397I) in GDF9 gene are associated with litter size in cashmere goats. Theriogenology.

[8] Ren F., Yu S., Chen R., Lv X., Pan C. (2017). Identification of a novel 12-bp insertion/deletion (indel) of iPS-related Oct4 gene and its association with reproductive traits in male piglets. Anim. Reprod. Sci..

[9] Baccetti B., Collodel G., Estenoz M., Manca D., Moretti E., Piomboni P. (2005). Gene deletions in an infertile man with sperm fibrous sheath dysplasia. Hum. Reprod..

[10] Coutton C., Escoffier J., Martinez G., Arnoult C., Ray P.F. (2015). Teratozoospermia: Spotlight on the main genetic actors in the human. Hum. Reprod. Update.

[11] Yang X., Zhu D., Zhang H., Jiang Y., Hu X., Geng D., Wang R., Liu R. (2018). Associations between DNAH1 gene polymorphisms and male infertility: A retrospective study. Medicine.

[12] Shen Y., Zhang F., Li F., Jiang X., Yang Y., Li X., Li W., Wang X., Cheng J., Liu M. (2019). Loss-of-function mutations in QRICH2 cause male infertility with multiple morphological abnormalities of the sperm flagella. Nat. Commun..

[13] Ben Khelifa M., Coutton C., Zouari R., Karaouzène T., Rendu J., Bidart M., Yassine S., Pierre V., Delaroche J., Hennebicq S. (2014). Mutations in DNAH1, which encodes an inner arm heavy chain dynein, lead to male infertility from multiple morphological abnormalities of the sperm flagella. Am. J. Hum. Genet..

[14] Merveille A.C., Davis E.E., Becker-Heck A., Legendre M., Amirav I., Bataille G., Belmont J., Beydon N., Billen F., Clément A. (2011). CCDC39 is required for assembly of inner dynein arms and the dynein regulatory complex and for normal ciliary motility in humans and dogs. Nat. Genet..

[15] Pausch H., Venhoranta H., Wurmser C., Hakala K., Iso-Touru T., Sironen A., Vingborg R.K., Lohi H., Söderquist L., Fries R. (2016). A frameshift mutation in ARMC3 is associated with a tail stump sperm defect in Swedish Red (Bos taurus) cattle. BMC Genet..

[16] Zariwala M.A., Omran H., Ferkol T.W. (2011). The emerging genetics of primary ciliary dyskinesia. Proc. Am. Thorac. Soc..

[17] Liu M., Huang S., Zhao X., Wu F., Zhu D., Zhai X., Wang A. (2021). Successful live birth following natural cycle oocyte retrieval in a woman with primary infertility and atypical primary ovarian insufficiency with a DNAH1 gene deletion mutation. Genet. Test. Mol. Biomarkers.

[18] Imtiaz F., Allam R., Ramzan K., Al-Sayed M. (2015). Variation in DNAH1 may contribute to primary ciliary dyskinesia. BMC Med. Genet..

[19] Emiralioğlu N., Taşkıran E.Z., Koşukcu C., Bilgiç E., Atilla P., Kaya B., Günaydın Ö., Yüzbaşıoğlu A., Tuğcu G.D., Ademhan D. (2020). Genotype and phenotype evaluation of patients with primary ciliary dyskinesia: First results from Turkey. Pediatr. Pulmonol..

[20] Wang Z., Pan Y., He L., Song X., Chen H., Pan C., Qu L., Zhu H., Lan X. (2020). Multiple morphological abnormalities of the sperm flagella (MMAF)-associated genes: The relationships between genetic variation and litter size in goats. Gene.

[21] Fagerberg L., Hallström B.M., Oksvold P., Kampf C., Djureinovic D., Odeberg J., Habuka M., Tahmasebpoor S., Danielsson A., Edlund K. (2014). Analysis of the human tissue-specific expression by genome-wide integration of transcriptomics and antibody-based proteomics. Mol. Cell Proteom..

[22] Wang Z., Zhang X., Jiang E., Yan H., Zhu H., Chen H., Liu J., Qu L., Pan C., Lan X. (2020). InDels within caprine IGF2BP1 intron 2 and the 3’-untranslated regions are associated with goat growth traits. Anim. Genet..

[23] Aljanabi S.M., Martinez I. (1997). Universal and rapid salt-extraction of high quality genomic DNA for PCR-based techniques. Nucleic. Acids Res..

[24] Müllenbach R., Lagoda P.J., Welter C. (1989). An efficient salt-chloroform extraction of DNA from blood and tissues. Trends Genet..

[25] Fontanesi L., Beretti F., Riggio V., Gómez González E., Dall’Olio S., Davoli R., Russo V., Portolano B. (2009). Copy number variation and missense mutations of the agouti signaling protein (ASIP) gene in goat breeds with different coat colors. Cytogenet. Genome Res..

[26] Nei M. (1973). Analysis of gene diversity in subdivided populations. Proc. Natl. Acad. Sci. USA.

[27] Reich D.E., Cargill M., Bolk S., Ireland J., Sabeti P.C., Richter D.J., Lavery T., Kouyoumjian R., Farhadian S.F., Ward R. (2001). Linkage disequilibrium in the human genome. Nature.

[28] Cui Y., Yan H., Wang K., Xu H., Zhang X., Zhu H., Liu J., Qu L., Lan X., Pan C. (2018). Insertion/Deletion within the KDM6A gene is significantly associated with litter size in goat. Front. Genet..

[29] Zhao H., Wu X., Cai H., Pan C., Lei C., Chen H., Lan X. (2013). Genetic variants and effects on milk traits of the caprine paired-like homeodomain transcription factor 2 (PITX2) gene in dairy goats. Gene.

[30] Jiang R., Cheng J., Cao X.K., Ma Y.L., Chaogetu B., Huang Y.Z., Lan X.Y., Lei C.Z., Hu L.Y., Chen H. (2019). Copy number variation of the SHE gene in sheep and its association with economic traits. Animals.

[31] Hui Y., Zhang Y., Wang K., Pan C., Chen H., Qu L., Song X., Lan X. (2020). Goat DNMT3B: An indel mutation detection, association analysis with litter size and mRNA expression in gonads. Theriogenology.

[32] Maiti A.K., Mattéi M.G., Jorissen M., Volz A., Zeigler A., Bouvagnet P. (2000). Identification, tissue specific expression, and chromosomal localisation of several human dynein heavy chain genes. Eur. J. Hum. Genet..

[33] Wambergue C., Zouari R., Fourati Ben Mustapha S., Martinez G., Devillard F., Hennebicq S., Satre V., Brouillet S., Halouani L., Marrakchi O. (2016). Patients with multiple morphological abnormalities of the sperm flagella due to DNAH1 mutations have a good prognosis following intracytoplasmic sperm injection. Hum. Reprod..

[34] Tu C., Nie H., Meng L., Wang W., Li H., Yuan S., Cheng D., He W., Liu G., Du J. (2020). Novel mutations in SPEF2 causing different defects between flagella and cilia bridge: The phenotypic link between MMAF and PCD. Hum. Genet..

[35] Zhuang B.-J., Xu S.-Y., Dong L., Zhang P.-H., Huang X.-P., Li G.-S., You Y.-D., Chen D., Yu X.-J., Chang D.-G. (2022). Novel DNAH1 Mutation Loci Lead to Multiple Morphological Abnormalities of the Sperm Flagella and Literature Review. World J. Men’s Heal..

[36] Cartharius K., Frech K., Grote K., Klocke B., Haltmeier M., Klingenhoff A., Frisch M., Bayerlein M., Werner T. (2005). MatInspector and beyond: Promoter analysis based on transcription factor binding sites. Bioinformatics.

[37] Kalkan G., Karakus N., Baş Y., Takçı Z., Ozuğuz P., Ateş O., Yigit S. (2013). The association between Interleukin (IL)-4 gene intron 3 VNTR polymorphism and alopecia areata (AA) in Turkish population. Gene.

[38] Vaz-Drago R., Custódio N., Carmo-Fonseca M. (2017). Deep intronic mutations and human disease. Hum. Genet..

[39] Xiang G., Ren J., Hai T., Fu R., Yu D., Wang J., Li W., Wang H., Zhou Q. (2018). Editing porcine IGF2 regulatory element improved meat production in Chinese Bama pigs. Cell Mol. Life Sci..

[40] Van Laere A.S., Nguyen M., Braunschweig M., Nezer C., Collette C., Moreau L., Archibald A.L., Haley C.S., Buys N., Tally M. (2003). A regulatory mutation in IGF2 causes a major QTL effect on muscle growth in the pig. Nature.

[41] Gibson J., Tapper W., Ennis S., Collins A. (2013). Exome-based linkage disequilibrium maps of individual genes: Functional clustering and relationship to disease. Hum. Genet..

[42] Lynch M., Xu S., Maruki T., Jiang X., Pfaffelhuber P., Haubold B. (2014). Genome-wide linkage-disequilibrium profiles from single individuals. Genetics.

