# Supplementary material for: Genetic Variations and mRNA Expression of Goat DNAH1 and Their Associations with Litter Size
Source: Cells. 2022 Apr 18;11(8):1371. doi: 10.3390/cells11081371 (PMC9024473; doi:10.3390/cells11081371)
Supplement: Supplementary file 1 [file cells-11-01371-s001.zip › cells-1648389-supplementary.pdf]

Table S1. PCR primers used for detecting in this study.

Primer	Loci	Sequences (5′-3′)	Length (bp)
SNP1	upstream variant	F: TTTTGTTGAGATGCCCTCCTTTAA<br>R: GTATAGGGATGTCTAGTCGGCCAC	911
SNP2	upstream variant	F: GGTGCTGGCTGACTGTGCTT<br>R: TGCCTACTGCCTCTTGGTACTCT	145/118
SNP3	upstream variant	F: GAAAGTGGGTGATGCTCTGGGA<br>R: TCATGGCATAATCCGGAACCAT	111
SNP4	upstream variant	F: GGGACTTGCTGACGAGACAA<br>R: GGTGGTACAAGACAGGGTTGG	143/128
SNP5	upstream variant	F: GGCAGGGCTTGGCAGTAAG<br>R: CCTCCCAGTTGGTCTCGTAGAG	241
SNP6	upstream variant	F: GAGGAGAAGGTGCCCAAGC<br>R: CAGGGAGGTCCCAGAGGAA	101
SNP7	upstream variant	F: GCCCTGACCAAGCCTGAACT<br>R: CAGAAGGGTGGGTGGGTAGA	102
SNP8	upstream variant	F: TGTTTGACTGGTACTTTACTTTT<br>R: TGTATGGCAAAACAAATATAATA	80
SNP9	upstream variant	F: AACTTGAATAACAGCTTGACTGG<br>R: ATACAAAGCAGGACAAGAGAACA	95
SNP10	upstream variant	F: GATGCTTTCCCGACGACC<br>R: GGTGCCCCCTACACAACG	113
CNV1	g:48593201-48595200	F: TGTCGGGAGTCAGCCCATA<br>R: GTGTCCCCTTCCCCTTACTG	100
CNV2	g:48603601-48605200	F: CAGTTGCCTCTCTGAGTCCC<br>R: AAACGGGCTTAGTGTGTCCC	133
CNV3	g:48617201-48618800	F: AGTGTCAGGAAATGGGGGTG<br>R: TGGTCCTGGCTATAACGGGT	166
qPCR-DNAH1	--	F: TGGCTGGGATATCACCCTGA<br>R: GTCACGCTCCAGGTCTTTCA	140
MC1R	--	F: GGCCTGAGAGGGGAATCACA<br>R: AGTGGGTCTCTGGATGGAGG	126
GAPDH	--	F: CCCGTTCGACAGATAGCCG<br>R: ACGATGTCCACTTTGCCAGT	143

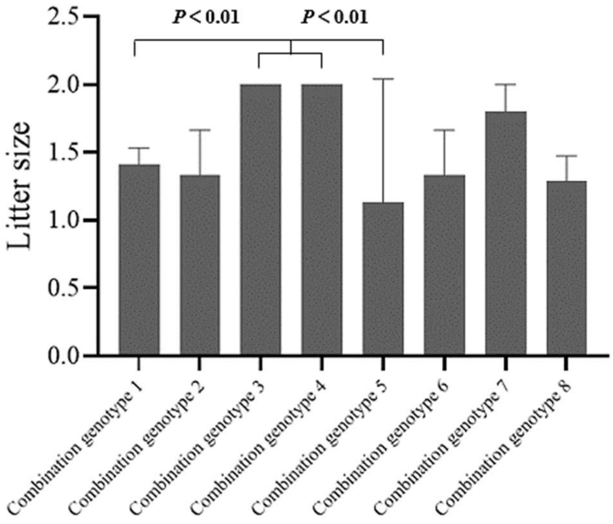

Figure S1. Relationship between the combined genotypes of SNP, the 27-bp InDel, and CNV variations within *DNAH1* gene and litter sizes in goats (mean ± standard errors). In the order of SNP1-SNP5-SNP7-InDel-CNV1-CNV2-CNV3. Combination genotype 1: AA-TT-CC-II-L_1-L_2-L_3, Combination genotype 2: AA-TT-CC-II-M_1-L_2-L_3, Combination genotype 3: AA-TT-CC-II-M_1-M_2-M_3, Combination genotype 4: AA-TT-CC-II-G_1-G_2-M_3, Combination genotype 5: AG-TA-CT-II- L_1-L_2-L_3, Combination genotype 6: AG-TA-CT-II-M_1-M_2-L_3, Combination genotype 7: AG-TA-CT-II- M_1-G_2-M_3, Combination genotype 8: GG-AA-TT-II-L_1-L_2-L_3.

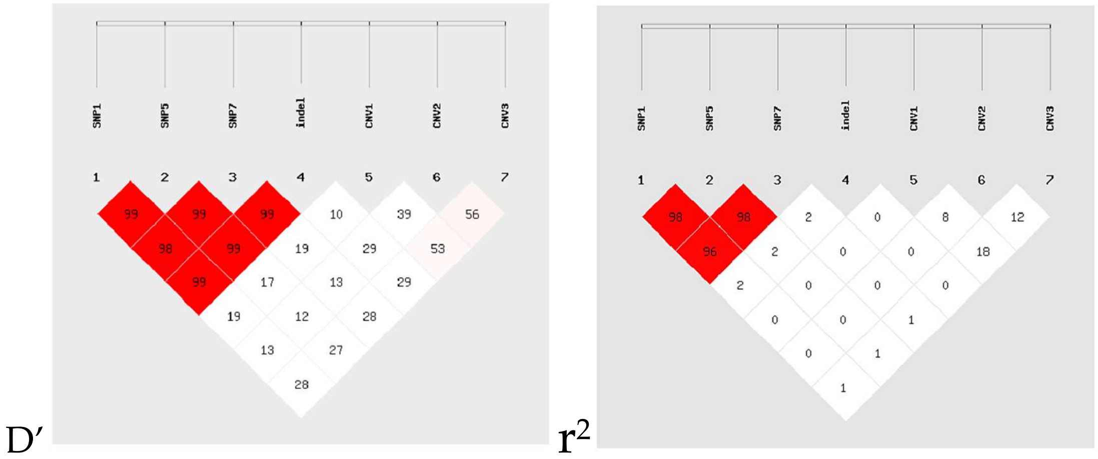

Figure S2. Linkage disequilibrium analysis of the SNP, InDel and CNV mutations within *DNAH1* in Shaanbei white cashmere goats. red = Loci are said to be in linkage disequilibrium.
